# Predictive clinical parameters for the response of nivolumab in pretreated advanced non-small-cell lung cancer

**DOI:** 10.18632/oncotarget.21602

**Published:** 2017-10-07

**Authors:** Yuko Oya, Tatsuya Yoshida, Hiroaki Kuroda, Masashi Mikubo, Chiaki Kondo, Junichi Shimizu, Yoshitsugu Horio, Yukinori Sakao, Toyoaki Hida, Yasushi Yatabe

**Affiliations:** ^1^ Department of Thoracic Oncology, Aichi Cancer Center Hospital, Nagoya, Japan; ^2^ Department of Thoracic Surgery, Aichi Cancer Center Hospital, Nagoya, Japan; ^3^ Department of Pathology and Molecular Diagnostics, Aichi Cancer Center Hospital, Nagoya, Japan

**Keywords:** nivolumab, non-small cell lung cancer, programmed cell death-1(PD-1), programmed cell death-ligand 1 (PD-L1)

## Abstract

**Background:**

Nivolumab offers a superior survival benefit over docetaxel in patients with advanced, previously treated non-small-cell lung cancer (NSCLC). An association between programmed cell death ligand-1 (PD-L1) expression and the efficacy of nivolumab has been reported in many studies. However, the association between the clinical parameters and efficacy of nivolumab remains unclear in advanced NSCLC patients.

**Results:**

Among 124 patients, 108 (88%) were performance status (PS) 0 to 1. PD-L1 expression was assessed in 89 patients, with 51 (57%) patients having PD-L1 positive expression. In all patients, the objective response rate (ORR) in patients with elevated CRP levels (≥ 1 mg/dl) was significantly worse than those without elevated CRP levels (< 1 mg/dl) (8.3 vs 23.4%, *p* = 0.0180). The PS (≥ 2), smoking index (< 400), CRP levels (≥ 1 mg/dl) and LDH (≥ 245 IU/L) were significantly associated with a shorter PFS and OS in patients treated with nivolumab. Multivariate analyses showed that the PS (≥ 2), smoking index (< 400), CRP levels (≥ 1 mg/dl) and LDH (≥ 245 IU/L) and PD-L1 expression were significant factors associated with a longer PFS of nivolumab.

**Materials and Methods:**

We retrospectively analyzed 124 patients who received nivolumab as a subsequent treatment. The patient characteristics, laboratory data at baseline (C-reactive protein [CRP] and lactate dehydrogenase [LDH]), PD-L1 expression, nivolumab response, progression-free survival (PFS), and overall survival (OS) were evaluated.

**Conclusions:**

Clinical parameters, such as PS, serum CRP, serum LDH, and smoking status, were significantly associated with the response duration and survival in patients treated with nivolumab.

## INTRODUCTION

Programmed cell death (PD)-1 immune checkpoint inhibitors have emerged as promising treatment options for multiple cancer types. These inhibitors bind with high affinity to the PD-1 receptors expressed on T cells and disrupt the negative signaling induced by PD-ligand 1 (PD-L1) and PD-L2 to restore T-cell effector function [1]. PD-1 inhibitors, nivolumab and pembrolizumab, were recently approved for treatment of advanced non-small-cell lung cancer (NSCLC). Nivolumab monotherapy showed a statistically superior survival benefit over docetaxel, which was standard therapy as a second line therapy in patients with advanced, previously treated NSCLC in two phase 3 studies [2–5]. The objective response rate (ORR) of nivolumab was approximately 15%, with the majority of responses persisting after treatment discontinuation in patients who stopped therapy for reasons other than disease progression [6, 7].

PD-L1 expression on tumor cells may be a possible predictive marker of a clinical response to anti-PD-1/PD-L1 antibodies. Indeed, the association of PD-L1 expression and the efficacy of them have been investigated in many studies [2, 3, 8, 9]. In phase III trials of nivolumab for previously treated non-squamous (SQ) NSCLC patients, PD-L1 expression was associated with the efficacy of nivolumab. In addition, first-line pembrolizumab has demonstrated significantly longer survival times compared to chemotherapy for PD-L1 expression strong positive NSCLC [10]. However, many patients with PD-L1 positive tumors do not respond to anti PD-1/PD-L1 antibodies, and some responses occur in patients with PD-L1 negative tumors [9]. Therefore, PD-L1 expression is not enough to predict the outcome to anti-PD-1/PD-L1 therapy. Recently, some biomarkers of PD-1 inhibitors and PD-L1 inhibitors efficacy, including high tumor mutational load [11, 12], neoantigen [11, 13], increased CD8 positive tumor-infiltrating lymphocytes in the tumor microenvironments [14], increased PD-L1 expression on immune cells [15, 16], and the presence of epidermal growth factor receptor (*EGFR*) mutation [17], have been reported. Unfortunately, these biomarkers are not entirely reliable and impractical. Reliable predictive markers that can be used to select patients with a higher likelihood of benefit from immune checkpoint inhibitors are needed. Additionally, some reports have suggested an association between clinical parameters, such as lactate dehydrogenase (LDH) and C-reactive protein (CRP), and the efficacy of immune checkpoint inhibitor in metastatic melanoma patients [18–21]. In contrast, there are few reports of the association between clinical parameters and the efficacy of anti-PD-1/PD-L1 therapy in advanced NSCLC patients. In addition, patients with poor performance status were not included in the anti-PD-1/PD-L1 antibodies trials. Therefore, we investigated various factors, including PD-L1 expression, on tumor, laboratory findings, and PS to identify predictive markers of nivolumab therapy in patients with NSCLC in this study.

## RESULTS

### Patients and treatment

**Figure 1:**
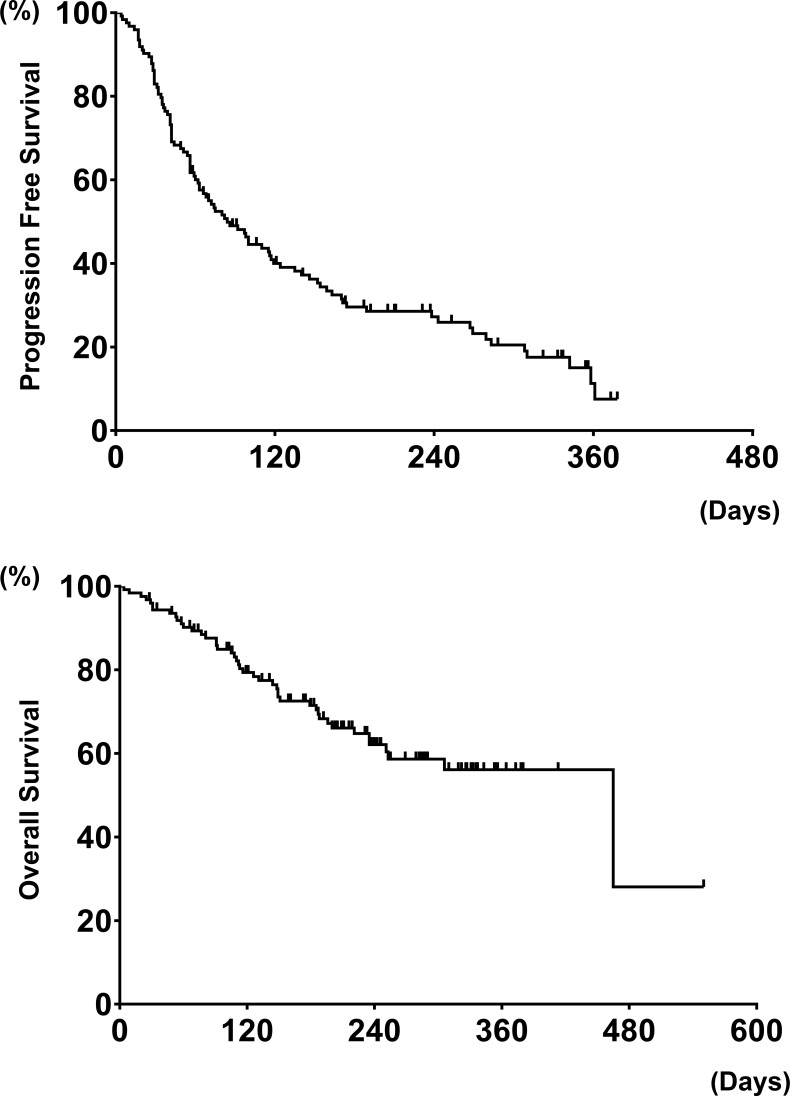
Progression free survival on nivolumab and overall survival from treatment with nivolumab in all patients (*N* = 124) (A and B)

The median follow-up was 6.0 months (range: 0.1 to 22.5 months). Patient characteristics are summarized in Table 1. The median age of the patients was 66 (range, 37–79) years. Eighty-one patients (65%) had adenocarcinoma (Ad), and seventy-seven (62%) patients were heavy smokers (smoking index [SI] ≥ 400). One hundred nine (88%) patients were ECOG PS score 0 to 1, with 15 (12%) patients at PS 2 to 3. All patients received cytotoxic chemotherapy previously, and 58 (47%) patients received nivolumab treatment as second line treatment (98% patients received platinum doublet chemotherapy). The median number of doses of nivolumab was five (range, 1 to 25). At the time of the database lock, 31% of the patients were continuing nivolumab treatment. Twenty-two patients (18%) harbored *EGFR* mutation, 14 (11%) had *KRAS* mutations, 5 (4%) had *HER2* mutation, 2 (2%) patients had *BRAF V600E* mutation, and no patients had *ALK* rearrangement. The median serum LDH and CR*P* values were 224 IU/L and 0.87 mg/dl. The LDH (≥ 245IU/L) and CRP (≥ 1.0 mg/dl) were elevated in 51 (41%) and 60 (48%) patients, respectively. For efficacy measurements, the ORR and median PFS in all patients were 16.1 % (95% confidence interval [CI]: 10.7–23.6) and 2.8 (95% CI: 2.1–4.0) months (Figure 1A), with the overall survival (OS) from treatment with nivolumab being 15.5 (95% CI: 8.3-not reached [NR]) months (Figure 1B).

**Table 1:** Patient characteristics (*N* = 124)

Characteristics		N	(%)
Age	Median [range]	66 (37–79)	
Sex	Male/Female	87/37	70/30
PS	0–1 2/3	109 12/3	88 10/2
Histology	Adenocarcinoma Squamous cell carcinoma Others	81 27 16	65 22 13
Smoking	Heavy Smoker (SI ≥ 400) Light smoker (0 < SI < 400) Never smoker (SI = 0)	77 20 27	62 16 22
Smoking index	Median [range]	630 [0–2520]	
Mutation	*EGFR* *KRAS* *HER2* *BRAF* None or Unknown	22 14 5 2 81	18 11 4 2 65
Treatment line	Second Third ≥ Fourth	58 20 46	47 16 37
PD-L1 expression	0 1–49 50– Unknown	38 42 9 35	31 34 7 28
LDH	< 245 IU/L ≥ 245 IU/L	73 51	59 41
CRP	< 1.0 mg/dl ≥ 1.0 mg/dl	64 60	52 48

Abbreviations: PS, performance status; SI; smoking index.

### Efficacy of nivolumab according to clinical parameters

**Figure 2:**
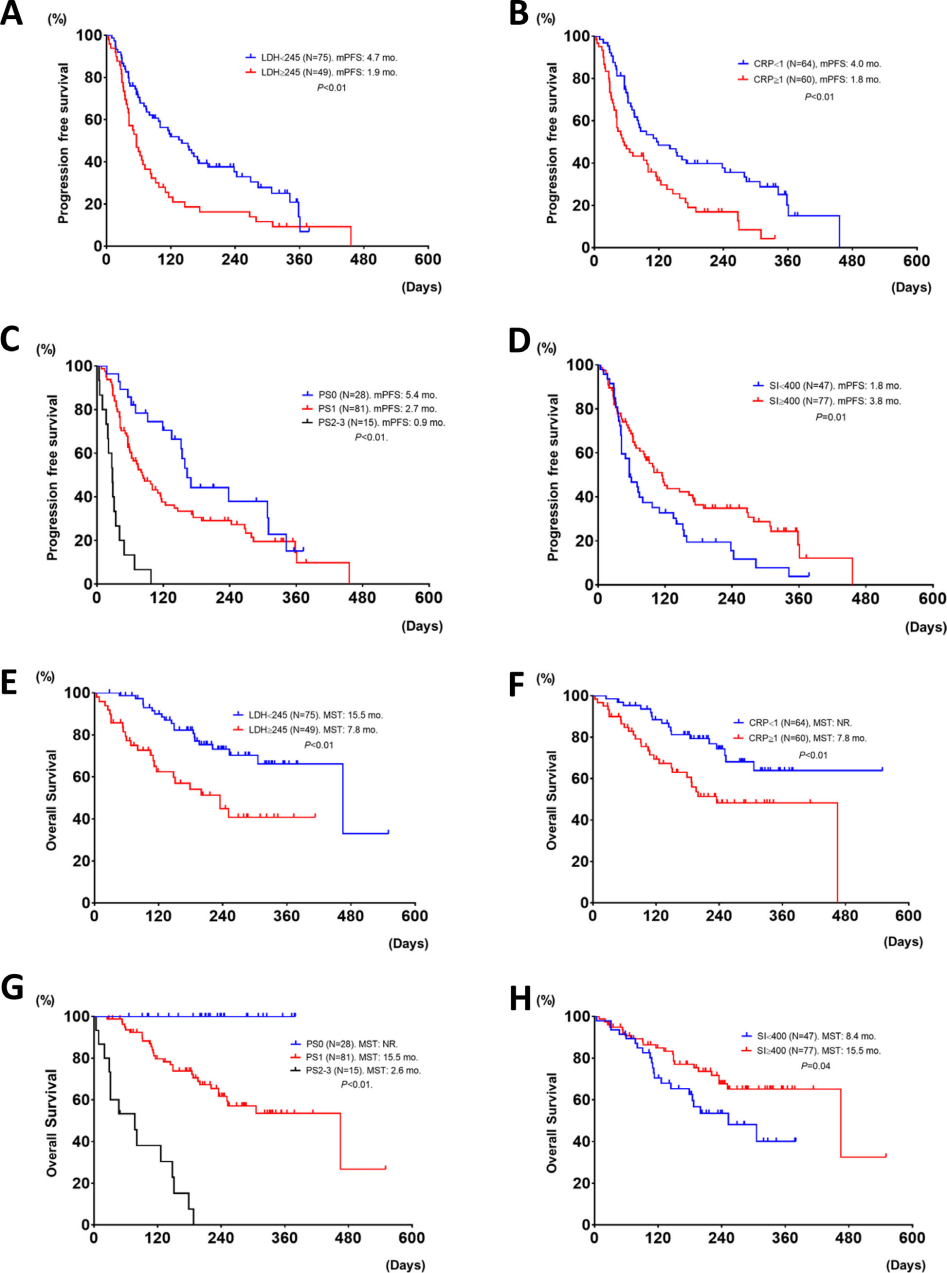
Progression free survival and overall survival in patients treated with nivolumab, based upon different LDH (≥ 245 vs. < 245), (**A** and **E**), CRP (≥ 1 vs. < 1), (**B** and **F**), PS (0 vs. 1 vs. 2), (**C** and **G**), and SI (≥ 400 vs. < 400), (**D** and **H**). mPFS; median progression free survival, mo; months, NR; not reached, and MST; median survival time.

The details of the ORR of nivolumab based upon the clinical parameters are shown in Table 2. The ORR of patients with elevated CRP levels was significantly worse that those without elevated CRP levels (8.3 vs 25.0%, *P =* 0.02). The PFS and OS in patients treated with nivolumab based upon clinical parameters is shown in Table 3. The PS, smoking index (SI), serum CR*P* values, and LDH values were significant factors for both PFS and OS in patients treated with nivolumab (Figure 2).

**Table 2:** Details on the efficacy of nivolumab based upon clinical parameters (*N* = 124)

Parameters			Response to Nivolumab, N (%)	*P*-value
Age	≥ 75 < 75	(*N* = 10) (*N* = 114)	1 (10.0) 20 (17.5)	> 0.99
Sex	Male Female	(*N* = 87) (*N* = 37)	16 (18.3) 5 (13.5)	0.79
SI	≥ 400 < 400	(*N* = 77) (*N* = 47)	17 (22.1) 4 (8.5)	0.08
PS	0 1 2–3	(*N* = 28) (*N* = 81) (*N* = 15)	7 (25.0) 14 (17.3) 0 (0.0)	0.10
Histology	Ad Sq Others	(*N* = 81) (*N* = 27) (*N* = 16)	9 (9.0) 7 (26.0) 5 (31.3)	0.05
LDH	≥ 245 IU/L < 245 IU/L	(*N* = 49) (*N* = 75)	8 (16.3) 13 (17.3)	> 0.99
CRP	≥ 1.0 mg/dl < 1.0 mg/dl	(*N* = 60) (*N* = 64)	5 (8.3) 16 (25.0)	0.02
Mutation status	*EGFR* *KRAS* Others *HER2* *BRAF* None/Unknown	(*N* = 22) (*N* = 14) (*N* = 7) (*N* = 5) (*N* = 2) (*N* = 81)	2 (9.0) 4 (28.5) 1 (14.2) 1 (20.0) 0 (0) 14 (17.2)	0.50

Abbreviations: SI, smoking index; Ad, adenocarcinoma; Sq, Squamous cell carcinoma.

**Table 3:** PFS and OS in patients treated with nivolumab based upon clinical parameters (*N* = 124)

Parameters			Median PFS Months (95% CI)	*P*-Value	MST Months (95% CI)	*P*-Value
Age	≥ 75 < 75	(*N* = 10) (*N* = 114)	2.7 (1.1-NR) 3.8 (2.0–3.9)	0.36	NR (7.9-NR) 15.5 (3.0–15.5)	0.34
Sex	Male Female	(*N* = 87) (*N* = 37)	3.3 (2.1–4.3) 2.3 (1.4–4.7)	0.59	15.5 (8.4–15.5) 10.2 (6.0-NR)	0.35
SI	≥ 400 < 400	(*N* = 77) (*N* = 47)	3.8 (2.5–5.7) 1.8 (1.4–3.2)	0.01	15.5 (15.5-NR) 8.4 (4.8-NR)	0.04
PS	0 1 2–3	(*N* = 28) (*N* = 81) (*N* = 15)	5.4 (4.0–10.3) 2.7 (2.0–3.9) 0.9 (0.3–1.1)	< 0.01	NR (NR-NR) 15.5 (7.8-NR) 2.6 (0.7–5.0)	< 0.01
Histology	Ad Sq Others	(*N* = 81) (*N* = 27) (*N* = 16)	2.4 (1.9–3.3) 4.9 (1.5–9.3) 3.2 (1.4-NR)	0.38	NR (8.4-NR) 15.5 (NR-NR) 7.8 (3.9-NR)	0.19
LDH	≥ 245 IU/L < 245 IU/L	(*N* = 49) (*N* = 75)	1.9 (1.3–2.7) 4.7 (2.6–6.3)	< 0.01	7.8 (3.9-NR) 15.5 (10.2-NR)	< 0.01
CRP	≥ 1.0 mg/dl < 1.0 mg/dl	(*N* = 60) (*N* = 64)	1.8 (1.4–3.3) 4.0 (1.9–7.9)	< 0.01	7.8 (5.0–15.5) NR (10.2-NR)	< 0.01
Mutation status	EGFR KRAS Others HER2 BRAF None/Unknown	(*N* = 22) (*N* = 14) (*N* = 7) (*N* = 5) (*N* = 2) (*N* = 81)	1.9 (1.2–5.1) 1.9 (0.9–3.9) 3.8 (1.2–4.7) 3.3 (1.3–5.7)	0.17	8.4 (4.2-NR) 6.6 (3.0-NR) NR (10.2-NR) 15.5 (3.7–15.5)	0.42

Abbreviations: MST; median survival time, NR; not reached.

### Association between efficacy of nivolumab and PD-L1 expression

**Figure 3:**
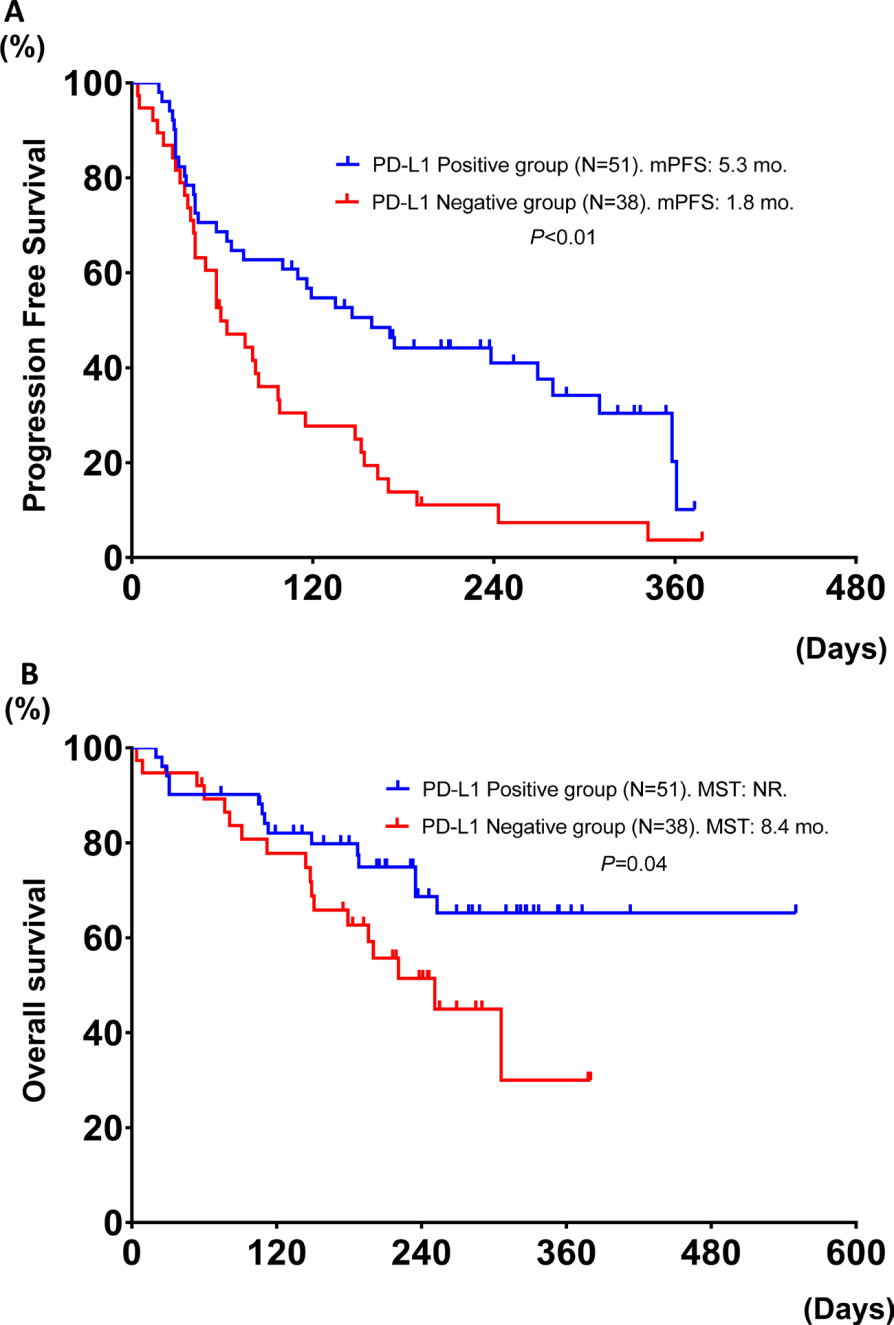
Progression free survival and overall survival in patients treated with nivolumab, based upon PD-L1 expression (positive vs. negative) (N = 89) (A and B)

In the 89 (72%) patients who had sufficient tumor tissues to evaluate quantifiable PD-L1 expression, 38 (43%) patients had PD-L1 negative expression, and 51 (57%) had PD-L1 positive expression, including 9 patients with more than 50% (≥ 50%) PD-L1 expression (Table 1). The ORR was significantly higher in patients with PD-L1 positive expression than those with PD-L1 negative expression (33% vs. 1.1%, *P <* 0.01). There was no difference in the ORR in patients with between 1–49% and ≥ 50% PD-L1 expression (33% vs. 33%, *P* > 0.99). The PFS and OS were significantly longer in patients with PD-L1 positive expression compared to those with PD-L1 negative expression (median PFS: 1.8 (95% CI: 1.4–2.8) months vs. 5.3 (95% CI: 2.2–9.3) months (Figure 3A), *P* < 0.01, and median OS: 8.4 (95% CI: 5.0-NR) months vs. NR (8.4-NR) months, *P =* 0.04) (Figure 3B).

### The multivariate analysis for the PFS of Nivolumab treatment

Multivariate analysis for PFS on nivolumab in 89 patients assessed by PD-L1 expression identified five factors associated with a longer PFS using nivolumab [LDH (< 245IU/L), HR: 0.55 (95% CI: 0.31–0.99), *P =* 0.04; CRP (< 1.0 mg/dl), HR: 0.48 (95% CI: 0.27–0.82), *P* = 0.01; PD-L1 expression positive, HR: 0.56 (95% CI: 0.33–0.98), *P* = 0.03; PS (0–1), HR: 0.42 (95% CI: 0.19–0.96), *P* = 0.04; and SI ≥ 400, HR: 0.53 (95% CI: 0.31–0.90), *P =* 0.02] (Table 4).

**Table 4:** The multivariate analysis of predictive factors for efficacy of Nivolumab (*N* = 89)

Variables	Multivariate analysis HR (95% CI) *P*-value	
LDH < 245 IU/L (vs. ≥ 245 IU/L)	0.55 (0.31–0.99)	0.04
CRP < 1.0 mg/dl (vs. ≥ 1.0 mg/dl)	0.48 (0.27–0.82)	0.01
PD-L1 expression Positive (vs. negative)	0.56 0.33–0.98	0.03
PS 0–1 (vs. 2–3)	0.42 (0.19–0.96)	0.04
SI ≥ 400 (vs. < 400)	0.53 (0.31–0.90)	0.02

Abbreviations: LDH: lactate dehydrogenase, CRP: C-reactive protein, SI: Smoking index, HR: hazard ratio.

## DISCUSSION

We found that PS, serum CRP and LDH values, smoking status, and PD-L1 expression were significantly associated with the PFS and survival in patients treated with nivolumab. Some reports have shown that PD-L1 expression and smoking status were predictive factors for the efficacy of nivolumab [2, 3, 8–10, 27, 28]. This is the first report to show that clinical parameters, such as serum CRP and LDH values, and PS are significantly associated with the response duration of nivolumab and survival in advanced NSCLC patients treated with nivolumab.

There have been some reports on the association between the clinical benefits of immune checkpoint inhibitors and clinical parameters, including laboratory data, in metastatic melanoma patients [18–21]. They showed that LDH at the baseline and changes in LDH correlate with response to another immune checkpoint inhibitor, ipilimumab, in melanoma patients [19, 29]. In addition, Diem et al. reported that serum LDH levels at baseline and during treatment could be a useful marker to predict the responsiveness or the progression in advanced melanoma patients who receive anti-PD-1 therapy [18]. LDH is the final enzyme in the glycolysis pathway that catalyzes interconversion of pyruvate and lactate. The gene expression and activity of LDH are often upregulated in cancer patients [30, 31]. High serum LDH levels have been linked to poor prognosis, which was consistent with our study [31–34]. In general, activated T cells, which are the key players in the anti-tumor responses of nivolumab, have been reported to use glycolysis as their main energy source [35]. As tumor cells release high amounts of lactate to the extracellular space, T-cells are not able to rid themselves of their own lactate. The extracellular lactate inactivates the cytokine release from dendritic cells and activated T-cells [36, 37]. These findings could mean that serum LDH levels are associated with the efficacy of immunotherapy, such as immune checkpoint inhibitors.

Our study also indicated that serum CRP levels are associated with the response to nivolumab. Serum CRP is one of the major acute-phase proteins, and is considered a definitive marker of systemic inflammation. In clinical practice, CRP is commonly used to evaluate the severity of systemic inflammation or outcomes of a variety of inflammation-related disorders. In the normal population, 70%–90% of samples have a CRP concentration of less than 0.3 mg/dl, while serum CRP levels in cancer patients are significantly higher and linked with tumor burden and disease progression [38]. Additionally, elevated serum CRP levels are associated with increased IL-6 production by tumor cells or by surrounding tissues. IL-6 has been reported to promote tumor-cell survival, and a higher level of IL-6 was significantly associated with an unfavorable prognosis in cancer patients [39, 40]. In this study, the reasons for the association with serum CRP levels and the efficacy of nivolumab remain unclear, but serum CRP and IL-6 levels have previously been shown to predict tumor response and survival to immunotherapy, such as high dose IL-2, IFN alpha, and ipilimumab [41–44]. Further investigation into the actions of IL-6 and CRP on immunotherapy efficacy is needed.

PS is a commonly used factor to determine the treatment and prognosis in patients with NSCLC. Advanced NSCLC patients with a poor PS (generally PS 3) do not benefit from standard chemotherapy [45]. In contrast, *EGFR* mutation-positive and ALK positive NSCLC patients with extremely poor PS often benefit from *EGFR*-TKIs and ALK-TKIs, which achieve high activity with acceptable toxicity levels in patients with a poor PS [46–48]. This study showed that the PS was a negative significant predictive factor for the efficacy of nivolumab. In our study, no patients with a PS 2–3 experienced a response to nivolumab, even if they have PD-L1 positive expression. The reasons for the lack of response in patients with a poor PS remain unclear, but the PS might reflect the immune state of patients and the tumor microenvironment. The immune system can perceive and eliminate some tumors early in their development. Based on the theory of immunoediting which involves the process of immunosurveillance, as tumors spread throughout the body, tumors can escape from the immune system through different mechanisms, such as alterations of reduced immune recognition, increased resistance to the cytotoxic effects of immunity, and the formation of an immunosuppressive state within the tumor microenvironment [49–51]. Therefore, the results of our study may suggest that the immune status of patients with poor a PS could be more immunosuppressive.

There were several limitations in this study. First, this was a retrospective study with a small sample size. Second, it is unclear whether the cutoff value for the LDH and CRP was relevant. Some reports have also shown that melanoma patients with an elevated baseline LDH had significantly shorter survivals compared to patients with a normal LDH treated with immune checkpoint inhibitors [18, 29]. There have been no reports on the differences in the efficacy of immune checkpoint inhibitors based upon different CRP levels. Thus, we analyzed the overall response rate of nivolumab based upon different CRP levels (Supplemental Table 1). Based on the distribution, a CRP of 1.0 mg/dl could be reasonable as the cutoff value. Thirdly, this study did not include information on AEs. In melanoma patients, AEs, including vitiligo and rash, were reported to be good prognostic factors for melanoma patients treated with nivolumab [52, 53]. Given these limitations, prospective trials will be required to confirm the impact of clinical parameters on the efficacy of immune checkpoint inhibitor treatments.

In conclusion, reliable predictive markers that can be used to select patients with a higher likelihood of benefit from immune checkpoint inhibitors remain unclear. Immune checkpoint inhibitors offer a new treatment for survival prolongation in advanced NSCLC patients. We found that PS and the levels of serum CRP and LDH values are not only prognostic factors, but also might be predictive factors for the PFS of treatment with nivolumab. This study might suggest that the efficacy of nivolumab likely depends on both tumor biomarkers and the patient status. Unlike treatment with *EGFR*-TKIs and *ALK*-TKIs, advanced NSCLC patients with high levels of serum CRP, serum LDH, and a poor PS might not be suitable for treatment with nivolumab. Further investigation including the efficacy of nivolumab in patients with a poor PS is warranted.

## MATERIALS AND METHODS

### Patients

We retrospectively analyzed 124 advanced, previously treated NSCLC patients who received nivolumab as subsequent treatment from January 2015 to January 2017 at the Aichi cancer center hospital. The patient characteristics, genetic characteristics (*EGFR*, v-Ki-ras2 Kirsten rat sarcoma viral oncogene homolog [*KRAS*], anaplastic lymphoma kinase [*ALK*], human epidermal growth factor receptor type 2[*HER2*], and *BRAF*), laboratory data at the baseline (CRP and LDH), PD-L1 expression of the tumor, nivolumab response, progression-free survival (PFS) of nivolumab, and overall survival (OS) were followed. Serum LDH and CRP levels were measured just before the initiation of treatment with nivolumab. The cutoff values for LDH and CRP were determined based upon standard values and previous reports [18, 22, 23]. For this study, serum lactate dehydrogenase (< 245 vs. ≥ 245 IU/L), and serum CRP (< 1.0 vs. ≥ 1.0 mg/dl) were the defined levels.

### Nivolumab treatment and response

Patients received at least one infusion of nivolumab (3 mg/kg every 2 weeks) as monotherapy. Patients continued this therapy until they showed progressive disease or experienced unacceptable adverse events. In general, patients underwent radiographic imaging every 2 months, and were evaluated for tumor response according to the response evaluation criteria in solid tumors, version 1.1 [24]. The ORR was calculated as the total percentage of patients with a complete response or a partial response.

### Mutation Analyses of *EGFR*, *ALK*, *KRAS*, *HER2*, and *BRAF*

*EGFR* mutations (exons 18–21) were identified using the cycleave polymerase chain reaction method. *HER2* (exon 20), *KRAS* (exons 2–3) and *BRAF* mutations (exons 11–15) were analyzed using fragment analysis, and the results were partially validated with direct sequencing, as previously reported [25]. *ALK* fusions were examined by reverse transcriptase PCR (RT-PCR), immunohistochemistry or fluorescence *in situ* hybridization assays (Vysis ALK Break Apart FISH Probe Kit; Vysis, Inc, Downers Grove, IL, USA), and a tumor was considered to be ALK positive when at least 2 of the RT-PCR, IHC, or FISH tests had positive results, as previously reported [26].

### PD-L1 expression analysis

Tumor PD-L1 protein expression was evaluated retrospectively in pretreatment (archival or recent) tumor-biopsy or surgical resection specimens with the use of an automated immunohistochemistry (IHC) assay (Dako, North America) that used rabbit monoclonal antihuman PD-L1 antibody (clone 28–8, Epitomics). Tumors were defined as PD-L1 positive when staining of the tumor-cell membrane (at any intensity) was observed at pre-specified expression levels of 1% or higher in a section that included at least 100 tumor cells for evaluation. In 124 patients, we identified 89 (72%) patients with tumor specimens that were evaluated for PD-L1 expression.

### Statistical analysis

All the statistical analyses were performed using the JMP version 11 statistical software package (SAS Institute, Cary, NC, USA). Differences in the baseline characteristics between the groups were compared using Fisher’s exact tests for categorical data. The PFS was calculated from the date of therapy initiation to disease progression. The OS was calculated from the date of nivolumab therapy initiation to death and censored at the date of last visit for patients whose death could not be confirmed. The survival probabilities were estimated using the Kaplan–Meier method, where differences in the variables were calculated using the log-rank test. Multivariate regression analysis was conducted according to the Cox proportional hazard model. Covariates with *P* ≤ 0.05 in the univariate analysis were included in the multivariate model. The database was locked on January 31th, 2017. At the time of the database lock, 44 of the 124 patients had died. This study was approved by the Institutional Review Board of the Aichi Cancer Center.

## SUPPLEMENTARY MATERIALS TABLE


