# Supplementary material for: Predictive clinical parameters for the response of nivolumab in pretreated advanced non-small-cell lung cancer
Source: Oncotarget. 2017 Oct 7;8(61):103117–28. doi: 10.18632/oncotarget.21602 (PMC5732716; doi:10.18632/oncotarget.21602)
Supplement: Supplementary file 1 [file oncotarget-08-103117-s001.pdf]

Predictive clinical parameters for the response of nivolumab in pretreated advanced non-small-cell lung cancer

SUPPLEMENTARY MATERIALS

Supplementary Table 1: The efficacy of Nivolumab according to different CRP levels ($N = 124$)

CRP levels	N	Response to Nivolumab N (%)
$0 \leq \times < 0.5$ mg/dl	($N = 49$)	10 (20.4)
$0.5 \leq \times < 1.0$ mg/dl	($N = 10$)	5 (33.3)
$1.0 \leq \times < 2.0$ mg/dl	($N = 19$)	1 (5.3)
$2.0 \leq \times < 3.0$ mg/dl	($N = 14$)	1 (7.1)
$3.0 \leq \times < 5.0$ mg/dl	($N = 10$)	1 (10.0)
$5.0 \leq \times$ mg/dl	($N = 17$)	2 (11.8)

CRP: C-reactive protein
